# You (bacteria) shall not pass: NLRP6 can sense you!

**DOI:** 10.1038/s44318-025-00638-3

**Published:** 2025-11-20

**Authors:** Alexander N R Weber, Philip Rosenstiel

**Affiliations:** 1 Institute of Immunology, Department of Innate Immunity, Auf der Morgenstelle 15, 72076 Tübingen, Germany; 2 CMFI – Cluster of Excellence (EXC 2124) “Controlling Microbes to Fight Infection”, University of Tübingen, Tübingen, Germany; 3 iFIT – Cluster of Excellence (EXC 2180) “Image-Guided and Functionally Instructed Tumor Therapies”, University of Tübingen, Tübingen, Germany; 4 German Cancer Consortium (DKTK) and German Cancer Research Center (DKFZ) Partner Site Tübingen, 72076 Tübingen, Germany; 5 Institute of Clinical Molecular Biology, Christian-Albrechts-University Kiel and University Hospital Schleswig-Holstein, Campus Kiel, 24105 Kiel, Germany

**Keywords:** Autophagy & Cell Death, Immunology, Microbiology, Virology & Host Pathogen Interaction

## Abstract

New research in *The EMBO Journal* explores the role of the NLRP6 inflammasome in sensing endolysosomal damage induced by pathogenic bacteria or other stimuli.

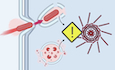

## Teaser

The ability of intracellular bacteria to spread directly from one cell to the next, avoiding extracellular immune defenses and drugs, is essential for pathogen dissemination and, ultimately, the progression and severity of infection. In this issue of *The EMBO Journal*, Boegli and colleagues uncover a previously unrecognized role of the enigmatic innate immune sensor NLRP6 in detecting bacteria-induced lysosomal damage and triggering cytokine release or cell death to limit infection.

Bacteria attempting to spread within a multicellular host face multiple challenges as they seek out protected niches that shield them from direct immune defense mechanisms. Extracellularly, they may be especially vulnerable to a whole arsenal of immune mediators, e.g., antimicrobial peptides, complement proteins, antibodies, or phagocytes. For invasive pathogens, including species of *Listeria, Shigella*, *Orientia, Chlamydia*, and *Salmonella*, a far safer route for spreading while avoiding the risks of the extracellular space, is direct cell-to-cell transmission from an infected host cell to its neighbor. For example, *Listeria* and *Shigella* propel themselves from the plasma membrane of an infected cell into the plasma membrane of an adjacent cell using the force generated by actin-based motility, and then become engulfed into a vacuole before escaping into the cytosol of the neighboring cell. However, the host has evolved an additional layer of defense involving a distinct family of cytosolic pattern-recognition receptors (PRRs) known as Nod-like receptors (NLRs). These intracellular sentinels can recognize specific microbe- or pathogen-associated molecular patterns (MAMPs or PAMPs, respectively), such as bacterial cell-wall components (e.g., muramyl dipeptide or flagellin), once these signals cross the membrane barrier and enter the cytosol. An alternative strategy of danger sensing by NLRs is the detection of cellular stress signals that reflect perturbations in homeostasis, e.g., potassium efflux, ROS, or ATP levels.

**Figure 1:**
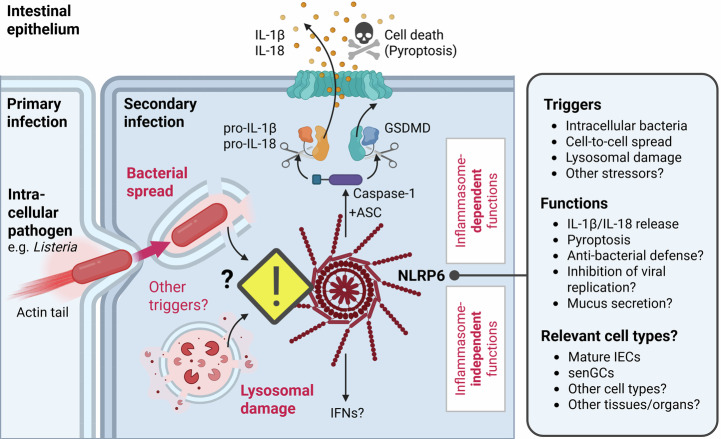
Sensing of bacterial escape and lysosomal damage by NLRP6. According to a new study in this issue of *The EMBO Journal* (Boegli et al, 2026), the enigmatic sensor NLRP6 appears to detect the cell-to-cell spread of pathogenic bacteria involving their escape from a cytosolic vacuole, which is mimicked by chemical perturbation, causing lysosomal damage. This results in the assembly and activation of an inflammasome, which leads to the maturation and release of pro-inflammatory cytokines (IL-1β and IL-18), as well as cell death. Rather than a specific microbe-derived molecule, NLRP6 can thus sense a potentially dangerous process or intestinal epithelial cellular integrity that can be associated with bacterial spread. It will be interesting to explore the extent to which an earlier described role, namely the transcriptional regulation of interferons (IFNs), is activated, and whether the novel mechanism reported here applies to other cell types, such as mucus-secreting sentinel goblet cells (senGCs), and/or other tissues with NLRP6 expression. Created with Biorender.com.

Although the family member NLRP6 had also been proposed to sense infections through recognition of specific MAMPs, Boegli et al now suggest that NLRP6 senses an infection-relevant *process* rather than a microbe-specific *molecule* (Fig. 1). Specifically, they show that NLRP6 can function as an intracellular “trip-wire” for incoming bacteria seeking to enter via a neighboring cell. It does so by assembling a so-called inflammasome, a multiprotein complex that bridges the sensor, NLRP6, to an enzyme, caspase-1, that is able to mature inflammatory cytokines (e.g., IL-1β or IL-18) for subsequent release, and to cleave gasdermin (GSDM) family proteins to execute pyroptotic, i.e., inflammatory, cell death. The effect is twofold: raising an alarm to infection and shutting down the bacterial hiding place.

The description of NLRP6 as a sensor for cell-to-cell spread was unexpected. NLRP6 (initially named PYPAF5) was first proposed not to act as a bona fide PRR but rather as a transcriptional regulator of other PRRs (Grenier et al, 2002) and a negative regulator of inflammatory signaling during bacterial pathogen clearance, including *Listeria* spp. (Anand et al, 2012). Only subsequently, NLRP6 was suggested to directly respond to certain metabolites (e.g., taurine, Levy et al, 2015) or a MAMP, namely double-stranded RNA from intestinal viruses (Wang et al, 2015), in intestinal epithelial cells (IECs), where it is most prominently expressed in both humans and mice. However, direct ligand binding and the underlying molecular mechanisms remained unclear until more recently, when RNA was shown to bind NLRP6 in vitro and trigger phase-separation as a potential way of bringing together a sufficient number of NLRP6 molecules to form an inflammasome (Shen et al, 2021). Apart from IECs (Levy et al, 2015), a role for NLRP6 was demonstrated in sentinel goblet cells (senGCs), specialized non-IEC cells that aid host defense via mucus production (Winsor et al, 2025). In SenGCs, NLRP6 was suggested to sense intestinal protozoa infection through monitoring microbial sphingolipids (Winsor et al, 2025). In myeloid immune cells, however, lipoteichoic acid (LTA), a cell-wall MAMP of certain gram-positive bacteria, was proposed to act as a direct NLRP6 agonist (Hara et al, 2018), highlighting the multi-faceted and/or controversial nature of NLRP6 sensing capabilities. An equally intense debate has centered on the physiological function of NLRP6 in the intestine. The initially reported putative role of NLRP6 in modulating microbiome composition, also in the context of colitis or tumorigenesis (e.g., Levy et al, 2015), was contested in subsequent studies (e.g., Mamantopoulos et al, 2017), illustrating the challenges of identifying bona fide roles of NLRP6 in a system as complex as the intestine. Besides the inherent challenges in microbiome studies, research on NLRP6 has also been hampered by the quality of available NLRP6 antibodies. It is therefore fair to say that defining the precise functions of NLRP6 has proven very difficult, let alone the roles of *human* NLRP6, given that practically all the above-mentioned studies focused on mice.

Compared to murine in vivo systems and structural studies, Boegli et al took an interesting cell-based approach by genetically modifying a human non-transformed intestinal epithelial cell line (HIEC-6) to express NLRP6. Interestingly, in their well-controlled system, all previously described purified MAMPs failed to trigger NLRP6 activity on their own. Viable bacteria were essential. Likewise, a *Listeria* mutant (*ΔactA*) that could no longer disseminate to neighboring cells (by ActA-dependent actin tails), even in combination with the aforementioned MAMPs, was unable to trigger NLRP6 activation compared to wild-type *Listeria*. Therefore, it was not primary HIEC cell infection alone that could trigger NLRP6, but rather spreading of bacteria to neighboring cells, and more specifically their escape from a double membrane vacuole into the cytosol during secondary infection. NLRP6 acting as a sensor for secondary infection was elegantly confirmed when uninfected, NLRP6-expressing HEK293T cells were seeded onto infected NLRP6-deficient HEK293T cells in antibiotic-containing media (which ruled out secondary infection via an extracellular route) and became activated. Thus, cell-to-cell spread involving endolysosomal destabilization emerged as essential for NLRP6 activation by *Listeria* and *Shigella*. Interestingly, NLRP6 was also activated by lysosome-destabilizing sterile treatments, identifying endolysosomal damage more generally as an NLRP6 activating process in this cellular system (Fig. 1). Perturbations of cellular homeostasis may thus represent the central pattern sensed by NLRP6 in intestinal epithelial cells, somewhat reminiscent of the “generalist” sensor role of NLRP3, the most prominent inflammasome sensor in myeloid and other immune cells (Weber et al, 2025), but which Boegli *et al*, ruled out in their IEC system. This article therefore proposes a novel and potentially very relevant new mechanism of sensing infection, but also sterile perturbations with relevance to a broad variety of pathologies including inflammatory bowel disease, where lysosomal damage has been proposed as a hallmark of chronic inflammation (Lassen et al, 2016).

Although mRNA and protein expression data seem to confirm NLRP6 as a major NLR in human intestinal cells, all the intestinal epithelial cell lines analyzed by the authors in a search for an endogenously NLRP6-competent cellular system failed to show significant NLRP6 expression. A significant limitation of the work consequently is that it was conducted exclusively in NLRP6-reconstituted systems. On the other hand, use of such defined systems adds clarity, as earlier studies using tissue explants could not always unequivocally attribute the observed effects to individual cell types, i.e., IECs, senGCs, or even infiltrating immune cells, each of which may respond to different signals and elicit different effector mechanisms. Although it remains to be demonstrated in vivo that bacterial spread alone—and not bacteria or their MAMPs— triggers NLRP6, the paper represents a significant advance as it provides a detailed functional characterization of NLRP6 in a human intestinal cell system and may prompt further analysis, e.g., of whether sensing of lysosomal integrity by NLRP6 contributes to homeostatic functions of senGCs. In addition, it would be interesting to establish to what extent NLRP6-dependent sensing of endolysosomal stability may be relevant beyond the intestine/infection contexts, but this will require similarly strict delineation (as presented here) from NLRP3.

Finally, although the study by Boegli et al (2026) focuses on inflammasome-related outcomes of NLRP6 activation, it is important to remember that one of the first roles ascribed to NLRP6 was transcriptional regulation (Wang et al, 2015), particularly of type-I interferons (IFN-I), through interaction with the viral sensor DHX15 and the adapter MAVS. An elegant study of a mouse model, in which two point mutations (R39E and W50E) prevent the inflammasome, but not the transcriptional regulatory function of NLRP6, recently showed that both inflammasome function and transcriptional regulation of IFN-I are important for full control of rotavirus infection (Li et al, 2024). Whether both are triggered by the same signal, here presumably direct RNA engagement (although *Boegli* and colleagues could not observe NLRP6 activation by RNA), awaits further exploration. Nonetheless, it would be interesting to determine whether the restriction of *Listeria* dissemination also involves transcriptional downstream mechanisms.

Key next steps will be to establish at the molecular level how exactly escaping *Listeria* cells “trip the wire” of NLRP6, and whether endolysosomal damage sets off multi-level inflammasome-dependent (and potentially also inflammasome-independent) signaling, also in chronic inflammatory diseases, such as human inflammatory bowel disease. Clearly, the challenge to more comprehensively understand the function of NLRP6 remains, and efforts in this direction should not only focus on the gut but also on other cell types (e.g., platelets) and organs (e.g., the brain), where interesting new evidence for a role of NLRP6 is accumulating.
